# Spectacle lenses with slightly aspherical lenslets for myopia control: clinical trial design and baseline data

**DOI:** 10.1186/s12886-022-02562-0

**Published:** 2022-08-16

**Authors:** Junhong Chen, Ran Zhuo, Jiayan Chen, Adeline Yang, Ee Woon Lim, Jinhua Bao, Björn Drobe, Daniel P. Spiegel, Hao Chen, Lijie Hou

**Affiliations:** 1 School of Ophthalmology and Optometry and Eye Hospital, Wenzhou Medical University, 270 Xueyuan West RoadZhejiang Province, Wenzhou, 310020 China; 2 Department of Optometry Center, Affiliated Eye Hospital of Wenzhou Medical University, Zhejiang Province, Hangzhou, China; 3 Essilor International Research Center (WEIRC), Wenzhou Medical University, Zhejiang Province, Wenzhou, China; 4 R&D AMERA, Essilor International, Singapore, Singapore; 5 Hangzhou Branch of Zhejiang Eye Hospital Affiliated to Wenzhou Medical University, 618 Fengqi East Road, Hangzhou, Zhejiang 310020 China

**Keywords:** Myopia, Spectacle lenses, Prospective studies, Refractive errors, Axial length

## Abstract

**Objectives:**

Myopia is a major public health problem and it is essential to find safe and effective means to control its progression. The study design and baseline data are presented for a one-year prospective, double-masked, crossover, randomized clinical trial evaluating the efficacy of single vision spectacle lenses with concentric rings of slightly aspherical contiguous lenslets technology (SAL) on myopia control.

**Methods:**

One hundred 8- to 13-year old Chinese children with a refractive error of -0.75 D to -4.75 D were assigned to two groups. In Group 1, SAL and single vision lenses were each worn for 6 months, and Group 2 wore the lenses in the reversed order. Primary outcomes are axial length and spherical equivalent of cycloplegic refractive error. Secondary outcomes included corneal thickness, anterior chamber depth, lens thickness, visual acuity, and lens adaptation.

**Results:**

No significant differences in baseline parameters (cycloplegic spherical equivalent, axial length, age) were found between groups (0.49 < *p* < 0.94). All children adapted well to the test lenses and there was no significant difference in visual acuity between the SAL and single vision lenses (*p* = 0.27).

**Conclusions:**

The children in the two well balanced groups had comparable visual acuity and adapted well to the test lenses. These results imply that visual acuity can be well improved by SAL lenses. Clear visual acuity provides the assurance for good compliance in this longitudinal study.

**Supplementary Information:**

The online version contains supplementary material available at 10.1186/s12886-022-02562-0.

## Background

Myopia has become a major public health problem worldwide. In the recentdecades, the prevalence of myopia has gradually increased. The prevalence of myopia and high myopia was 22.9% and 2.7% of the world population in 2000, and it is expected to rise to 49.8% and 9.8%, respectively in 2050 [1]. Compared with other regions, the prevalence of myopia is highest in Asia, especially in East Asia [2–4]. Lin et al. [3] reported that the prevalence of myopia in Taiwan rosefrom 74 to 84% from 1983 to 2000 in 16 to 18 year- old children. Furthermore, the prevalence of high myopia in 18-year-old students increased from 10.9% to 21%.

Excessive progression of myopia has been shown to be associated with sight-threatening complications [5–7]. The odds of complications increase with a higher degree of myopia and greater axial length [8]. Thus, preventing the progression of myopia and slowing down the elongation of the eye at an early stage is vital to avoid later ocular complications.

Several interventions are currently available to slow myopia progression, such as topical administration of atropine and use of orthokeratology (Ortho-K), multifocal contact lenses, or specially designed spectacle lenses. High doses of atropine are effective but associated with significant adverse effects such as photophobia and impaired accommodation. In addition, the significant rebound effect after cessation of high-concentration atropine limits its widespread use [9, 10]. Lower dosages of topical atropine (0.01%) have feweradverse effects; however, the axial length control effect is limited [11]. Ortho-K is effective in myopic refractive error and myopia control [12–14], but the efficacy is significantly associated with corneal shape [15–18]. Recently, many studies have reported that dual-and multifocal soft contact lenses are effective strategies for slowing myopia progression [19–22]. However, potential ocular complications such as conjunctivitis and keratitis [23] limit the large-scale usage of soft contact lenses in myopia control.

On the other hand, spectacle lenses represent a safe and easy-to-administer option for myopia control. Among spectacle lenses, (prismatic) bifocals provide the best myopia control efficacy [24]; however, aesthetics considerations hinder their more extensive usage. Progressive addition spectacle lenses (PALs) are more aesthetic, but their treatment effect is smaller [25–28]. Spectacle lenses with peripheral hyperopic defocus amelioration have minimal, if any, myopia control effects [29, 30]. Recently, introduced spectacle lenses with myopia control segments may be a promising avenue in myopia control combining good efficacy and aesthetics [31, 32].

Hence, a randomized double masked crossoverclinical trial was undertaken to evaluate the myopia control effect of novel spectacle lenses with contiguous slightly aspherical lenslets (SAL).

## Methods

### Study design

**Fig. 1:**
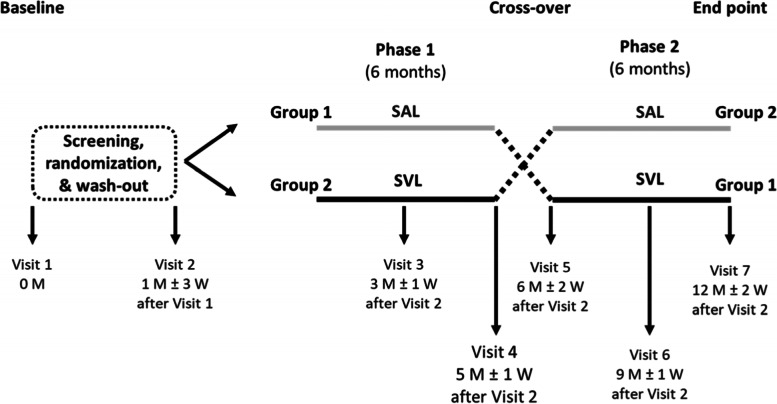
Flow chart of the study; M – month(s), W – week(s)

This is a prospective, double-masked, crossover, and randomized clinical trial. The duration of the study is 13 months (see Fig. 1 for more details about the timeline of this study), and it consists of 7 visits.

Eligible subjects were randomized into two groups. Group 1 will wear SAL lenses for 6 months, followed by 6 months of wearing single vision lenses. In Group 2, the order in which the lenses are worn is reversed. In this paper, we refer to the spectacles worn during the first 6 months (regardless of lens type) as study equipment 1 and the spectacles worn during the second 6 months (regardless of lens type) as study equipment 2 (Fig. 1). At each visit, all subjects were instructed to wear the study equipment for more than seven hours every day.

The study is being conducted at the Hangzhou Branch of Zhejiang Eye Hospital Affiliated to Wenzhou Medical University. The study and its protocol followed the tenets of the Declaration of Helsinki and were approved by the institutional review board of the Eye Hospital of Wenzhou Medical University. Written informed consent was obtained from participants and their guardians after a detailed explanation of this study and the possible risks and benefits at the first visit. The study is registered at the Chinese Clinical Trial Registry (ChiCTR1900021002, 24/01/2019).

### Study participants

A total of 100 children were recruited from the hospital into this study between January and March 2019. The inclusion criteria were best-corrected visual acuity equal to or better than 1.0, age between 8 and 13 years inclusive, spherical equivalent of cycloplegic autorefraction between -0.75 D to -4.75 D in each eye, astigmatism no more than 1.50 D, anisometropia no more than 1.00 D, and absence of strabismus. Subjects with a history of ocular or systemic disease, history of wearing PALs, or prior myopia control treatment were excluded from participation in the study.

The study was designed to achieve 80% power to detect a minimum difference of 0.27 D with an SD of 0.37 D at 6 months at a 5% level of significance. Using an online sample size calculator by the Clinical & Translational Science Institute33, the theoretical sample size was 42 with a 1:1 sample ratio in each group. Allowing a maximum drop-out rate of 15%, the number to be recruited for each group was estimated to be 50.

### Randomization and masking

All subjects were assigned into the two groups (described in the Study Design section) at a 1:1 ratio using covariate-adaptive randomization [33]. A scheduled randomization was generated and children were randomly assigned by Study Manager (ESSILOR R&D). The investigators cannot accessthe randomization list.

According to the scope of responsibilities, the examiners are either masked or unmasked. The masked examiners are responsible for subjective refraction, cycloplegic autorefraction, axial length, corneal thickness, anterior chamber depth measurements and assessing visual acuity with the study equipment. The unmasked examiners are in charge of dispensing and documenting adverse events.

### Test lenses

The treatment lens is a polycarbonate single vision spectacle lens with a spherical front surface with 11 concentric rings formed by contiguous slightly aspherical lenslets.

The control lens is a standard polycarbonate single vision lens.

### Main outcome measures

There are two main outcome measures: the axial length (AL) and the spherical equivalent (SER) of cycloplegic autorefraction. The AL is measured using an optical low-coherence reflectometry device (Lenstar LS900; Haag-Streit, Koeniz, Switzerland). Five measurements were taken, and the average value per eye was used for data analysis. The refraction is measured by closed-field autorefractors (Topcon KR-800 and Topcon 8900). The measurement was taken ten times on each eye and the mode of the sphere, cylinder, and axis per eye is recorded for data analysis. The SER is calculated as sphere + 0.5 × cylinder. The SER is taken only at baseline, the fifth visit and the last visit to minimize cycloplegia during the study.

### Secondary outcome measures

Corneal thickness (CT), anterior chamber depth (ACD), and lens thickness (LT) were measuredwith a Lenstar LS900 (Haag-Streit, Koeniz, Switzerland) together with AL. Five measurements were taken, and the average value per eye was used for further data analysis. Distance-corrected visual acuity (DCVA) is evaluated using a standard 100% contrast English Early Treatment Diabetic Retinopathy Study (ETDRS) logMAR chart at 4 m with usual correction or new distance prescription. DCVA is scored using the standard technique of subtracting 0.02 logMAR units for each correctly identified optotype.

### Study equipment questionnaire

The questionnaire contains six questions. The first four questions evaluate adaptation and subjectively evaluate various aspects of vision with the study equipment on a scale of 1 to 10. Question 1 quantifies the clarity of vision (1 = blurred, 10 = clear); question 2 evaluates the perception of ghost images (1 = none, 10 = severe); question 3 evaluates satisfaction with the study equipment (1 = not satisfied, 10 = satisfied), and question 4 evaluates the comfort of the study equipment (1 = uncomfortable, 10 = comfortable). The remaining questions assess compliance in terms of how long the study equipment is worn (question 5: hours per day, and question 6: days per week).

### Study visits and procedures

The procedures performed at each visit are summarized in Table 1.

**Table 1:** Visit schedule for the study. V1 to V7 represent visits 1 through 7. AL Axial length, *CT* Corneal thickness, *ACD* Anterior chamber depth, *LT* Lens thickness, *DCVA* Distance-corrected visual acuity

Visit schedule	V1	V2	V3	V4	V5	V6	V7
Informed consent	X						
Eye examination	X						
Inclusion and exclusion criteria	X						
Study frame choice	X						
Monocular pupillary distance and fitting measurements	X			X			
Washout spectacles delivery	X						
Study spectacles delivery		X			X		
AL, CT, ACD and LT measurements	X	X	X	X	X	X	X
Cycloplegic autorefraction	X				X		X
Non-cycloplegic subjective refraction	X			X			
DCVA	X	X	X	X	X	X	X

During the recruitment and baseline visit (V1), we first performed a full ocular examination, including presenting visual acuity, objective and subjective refraction, anterior segment and fundus inspection, and binocular vision status (near and far phoria, near point of convergence (NPC), Worth 4-dot test), followed by acquisition of the primary outcome measures (cycloplegic SER and AL) and all secondary outcome measures (DCVA, AL, CT, ACD, and LT). The subjects chose their frame, and the same frame was used for SAL and control single vision lenses, i.e., before and aftercrossover. At the end of the baseline visit, the subjects are dispensed with washout single vision lenses corresponding to the prescription for their first study equipment.

The main follow-up visits included the 6-month crossovervisit (V5) and the final 12-month visit (V7) with all primary outcome measures, including the questionnaires.

The additional study visits comprise the dispensing visit (approximately one month after the baseline, V2), 3-month (V3), 5-month (V4), and 9-month visits (V6) with only noncycloplegicAL and secondary outcome measures. In addition, at the 5-month visit, we acquired noncycloplegicsubjective refraction as a basis for the prescription for the study equipment worn during the second phase of the study (i.e., aftercrossover). Before ordering a new prescription, trial frame was used to confirm comfort of each participant.

Between one and three days after the dispensing visit and 6-month visit, we administered the compliance and adaptation questionnaire over the phone.

During all study visits, the study equipment was adjusted, and subjects were instructed to wear it at least 7 h per day.

Cycloplegia is always induced by 1% cyclopentolate hydrochloride (Alcon Laboratories) eyedrops delivered three times, five minutes apart after induction of corneal anaesthesia with proparacaine (0.5% Alcaine, Alcon Laboratories).

### Data analyses

#### Baseline data analyses

Paired-sample t testwere used for within-subject interocular comparisons and independent-sample t test- were used for between-group comparisons. Bivariate correlations were used to evaluate the relationships between outcome measures. Sincethe -intereye correlation for ocular parameters was high, only the right eye data wereanalysed. Statistical analyses were performed using SPSS 16.0 (SPSS Inc., Chicago, IL, USA).

#### Prospective data analyses

The changes in spherical equivalent cycloplegic autorefractiveerror and AL between the follow-up and baseline data will be used to evaluate myopia progression. Myopia progression and axial elongation will be compared between the SAL lenses and the single vision lenses using independent-samplet test. A multivariate regression model will be used to evaluate the relationships between variables such as age, gender, baseline myopia, parental myopia, NPC and phoria level, and myopia progression.

## Results

### Overall baseline data

We recruited 102 children from the Hangzhou Branch of Zhejiang Eye Hospital. One child dropped out just after the randomization period for personal reasons. One child was excluded from the study because of his or herlarge magnitude of phoria that manifested before the dispensing visit. Thus, the data from 100 children (54 males and 46 females) aged 8 to 12 years old (mean age, 9.49 ± 1.42 years) were analysed and are reported in this paper. We found no significant difference in the SER or AL between the eyes (t = 1.11, *p* = 0.27, 95% CI: -0.03659, 0.12879 and t = -1.04, *p* = 0.30, 95% CI: -0.05951, 0.01867 respectively).

The mean AL was 24.84 ± 0.76 mm (range: 23.13 to 26.86 mm), and the mean SER was -2.69 ± 0.86 D (range: -1.00 to -4.50 D). These two measures were significantly correlated (r = -0.432, *p* < 0.001).

More details on the baseline values, including the secondary outcome measures, can be found in Table 2.

**Table 2:** Baseline characteristics of the subjects and interocular comparison. Data are expressed as the mean ± *SD*. *SER*, Spherical equivalent refractive error, *AL* Axial length, *CCT* Central corneal thickness, *ACD* Anterior chamber depth, *LT* Lens thickness, t/p, paired-sample t-tests were used to compare the values between the two eyes

	Age (years)	SER (D)		AL (mm)		CCT (µm)		ACD (mm)		LT (mm)	
		OD	OS	OD	OS	OD	OS	OD	OS	OD	OS
Minimum value	8	-4.50	-4.75	23.13	22.84	473.40	480.00	2.76	2.73	2.97	3.00
Maximum value	12	-1.00	-1.13	26.86	27.05	603.60	614.80	3.80	3.77	3.61	3.65
Mean value	9.49 ± 1.42	-2.69 ± 0.86	-2.74 ± 0.94	24.84 ± 0.76	24.86 ± 0.76	545.20 ± 26.78	546.02 ± 27.61	3.25 ± 0.20	3.25 ± 0.20	3.32 ± 0.15	3.32 ± 0.15
Differences between two eyes	-	0.33 ± 0.26		0.16 ± 0.12		3.80 ± 4.57		0.03 ± 0.03		0.02 ± 0.03	
t value	-	1.11		-1.04		-1.39		0.46		-1.23	
*p* value	-	0.27		0.30		0.17		0.65		0.22	

### Between-group comparison of baseline data

Eligible subjects were randomized to two groups of 50 subjects. The mean age was 9.48 ± 1.36 years (range: 8 to 12) in Group 1 and 9.50 ± 1.50 years (range: 8 to 12) in Group 2. There was no significant difference between the groups (t = -0.07, *p* = 0.94).

**Fig. 2:**
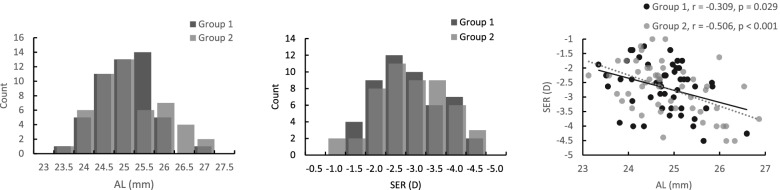
Distributions and relationships between the primary outcome measures at baseline. Left panel – axial length (AL), middle panel – cycloplegic spherical equivalent (SER), right panel – correlations between AL and SER. Lighter shade shows Group 1 (SAL); darker shade Group 2 (single vision lens; SVL). Triangles filled with the corresponding shade indicate the groups’ means

The two groups were matched for SER and AL. The mean AL was 24.79 ± 0.67 mm (range: 23.35 to 26.59 mm) in Group 1 and 24.89 ± 0.84 mm (range: 23.13 to 26.86 mm) in Group 2. The mean SER was -2.68 ± 0.83 D (range: -1.25 to -4.50 D) in Group 1 and -2.72 ± 0.90 D (range: -1.00 to -4.50 D) in Group 2. There was no significant difference between the groups for AL or SER (t = -0.69, *p* = 0.49 and t = 0.22, *p* = 0.83, respectively). SER and AL were negatively correlated in both groups (Group 1:r = -0.309, p = 0.029; Group 2:r = -0.506, *p* < 0.001; Fig. 2).

There were no significant differences between the groups for any of the secondary outcome measures (-0.88 < t < 1.06, 0.29 < *p* < 0.80). More details on the baseline values in the two groups, including the secondary outcome measures, can be found in Table 3.

**Table 3:** Characteristics of the subjects by group. Data are expressed as the mean ± *SD*. *SER* Spherical equivalent refractive error, *AL* Axial length, *CCT* Central corneal thickness, *ACD* Anterior chamber depth, *LT* Lens thickness, t/p, independent-sample t-tests were used to compare between-group parameters; G1, Group 1; G2, Group 2

	Age (y)		Gender		SER (D)		AL (mm)		CCT (µm)		ACD (mm)		LT (mm)	
	G1 G2		G1 G2		G1 G2		G1 G2		G1 G2		G1 G2		G1 G2	
Boys			27	27										
Girls			23	23										
Minimum value	8	8			-4.50	-4.50	23.35	23.13	473.4	505.4	2.86	2.76	2.97	3.05
Maximum value	12	12			-1.25	-1.00	26.59	26.86	603.0	603.6	3.80	3.76	3.60	3.61
Mean value	9.48 ± 1.36	9.50 ± 1.50			-2.68 ± 0.83	-2.72 ± 0.90	24.79 ± 0.67	24.89 ± 0.84	545.88 ± 27.99	544.53 ± 25.78	3.28 ± 0.22	3.23 ± 0.19	3.31 ± 0.16	3.33 ± 0.14
t value	-0.07				0.22		-0.69		0.25		1.06		-0.88	
*p* value	0.94				0.83		0.49		0.80		0.29		0.38	

### Lens adaptation

All subjects adapted well to both SAL lenses and single vision lenses (Table 4). The mean DCVA at the dispensing visit was 0.06 ± 0.08 logMAR (range: -0.14 to 0.24 logMAR) in Group 1 (SAL lenses) and 0.04 ± 0.05 logMAR (range: -0.1 to 0.14 logMAR) in Group 2 (single vision lenses, SVL), with no significant difference between the groups (t = 1.11, *p* = 0.27).

**Table 4:** Adaptation to the SAL and single vision lenses. *DCVA* Distance-corrected visual acuity. Data expressed as the mean ± SD. Group 1: in phase one for Group 1, the subjects wear the SAL lenses; Group 2: in phase one for Group 2, the subjects wear single vision lenses (SVL)

	SAL	SVL	t value	*p* value
DCVA	0.06 ± 0.08	0.04 ± 0.06	1.11	0.27
Perception of ghost images	1.04 ± 0.20	1.00 ± 0.00	1.43	0.16
Comfort of spectacles	9.68 ± 0.55	9.84 ± 0.42	-1.64	0.11
Clarity of vision	9.70 ± 0.58	9.93 ± 0.25	-2.59	0.01
Vision satisfaction	9.72 ± 0.50	9.91 ± 0.29	-2.32	0.02

For the perception of ghost images, we found no significant difference between the lens types (mean values: 1.04 ± 0.20 for SAL lenses and 1.00 ± 0.00 for SVL lenses; t = 1.43, *p* = 0.16). Similarly, there was no significant difference in the comfort of the spectacles (mean values: 9.68 ± 0.55 for SAL lenses and 9.84 ± 0.42 for SVL lenses; t = -1.64, *p* = 0.11). Regarding clarity of vision and vision satisfaction, small but significant differences were observed between the two lens types. The subjects rated single vision lenses as providing higher clarity of vision (mean values: 9.70 ± 0.58 for SAL lenses and 9.93 ± 0.25 for SVL lenses; t = -2.59, *p* = 0.01) and vision satisfaction (mean values: 9.72 ± 0.50 for SAL lenses and 9.91 ± 0.29 for SVL lenses: 0.19; t = -2.32, *p* = 0.02).

### Family history and supplementary information

The average height of the participants was 142.51 ± 11.01 cm, and the mean value of weight was 35.21 ± 9.09 kg. According to the feedback from guardians, 72% of fathers and 76% of mothers had myopia. Among the 100 families, both parents had myopia, accounting for 58%, and only 10% had nonmyopic parents.Most parents graduated from university and above (81% for fathers and 83% for mothers). Only 7% of fathers and mothers have a secondary school degree or less. Of the 100 participants, 73 reported that their first correction of myopia was 8.41 ± 1.54 years, and the remaining 27 participants reported that they had never found or corrected myopia before. Forty—six participants had provided personal information about hobbies. Based on the responses from participants, hobbies were divided into three categories: outdoor activities, indoor activities and near work activities. The percentages for each category were 15%, 22% and 63% respectively. The most commonly reported activities were near work activities, including playing piano/guitar/lego/guzheng/chess, reading, drawing and watching television. This was followed by indoor activities, including swimming, dancing, singing, skating, taekwondo and playing ping pong. The outdoor activities included playing dadminton/soccer, skiing and bicycle riding.

## Discussion

Although numerous interventions are currently available to slow myopia progression, it still be beneficialfor young children to develop effective and safe strategies for myopic retardation. Spectacle lenses with contiguous aspherical lenslets may be an interesting option. At present, there are few reports on the myopia control effect of these lenses. From this perspective, we conducted a double-masked, crossover, randomized clinical trial to assess the efficacy of spectacle lenses with rings of contiguous slightly aspherical lenslets.

We followed 100 children randomized into two groups of 50. The two groups were well matched for the main covariate parameters, such as age, spherical equivalent refractive error, and AL (0.49 < p < 0.94), minimizing their effects on the results of the study.

In this study, we adopted a one-year crossoverdesign that is not very common in myopia research. We believe that this approach has merit, particularly for completely novel interventions whose myopia control effect is difficult to infer from previous studies. Unlike regular clinical trial design, this crossoverdesign may provide a within-subject treatment comparison with little to no influence of genetics and other subject-specific factors. However, it must be noted that these analyses should be interpreted with caution because they may be biased by possible carry-over effects or difficulty with subject masking.

We also opted to have a shorter interval (maximum of 3 months) between the study visits; that is, a maximum of 3 months versus the more standard 6 months. This decision was motivated by the interest in evaluating the myopia control effect of SAL lenses at finer temporal resolution. Although cycloplegic SER and AL are only taken at baseline, and at the 6- and 12-month visits to minimize cycloplegia, we believe that these data will provide valid evidence for the effect of myopia control.Moreover, AL without cycloplegia will be taken at the 3-, 5-, and 9-month visits, and these data will be informative. First, the AL is considered animportant index to evaluate myopic progression [34, 35]. Second, previous research has indicated that (lack of) cycloplegia has no significant effect on AL [36]. In addition, 3-month visits may allow better compliance through parent and child engagement and, more often, equipment checks.

In general, the children adapted well to both SAL and single vision lenses. According to the results of the questionnaire, there were some small but statistically significant differences in the clarity of vision and vision satisfaction between the two spectacle lens types. Although children rated the clarity of vision and overall visual satisfaction higher with SVL, the SAL lenses did not aggravate the severity of blur or induce higher discomfort. Moreover, the mean values were above 9 in both groups and the differences were 0.23 for clarity of vision and 0.19 for vision satisfaction. Considering that the values came from a questionnaire with a 1 to 10 score, these differences are likely clinically irrelevant.

According to spontaneous verbal feedback of SAL group wearers, five of the children reported slight perception of blur in the peripheral vision, and two children reported blur when reading, but they adopted a strategy to obtain a clear image. One child felt dizzy on the first day of using the SAL lens. In all these children, the adaptation did not exceed one day, and none of them discontinued the trial because of an adaptation problem.

To ensure the accuracy of the DCVA, measurements were taken using an ETDRS logMAR chart at 4 m under stable room lighting conditions. Although the corrected visual acuity was slightly better with single vision lenses, we did not find a significant difference between SAL lenses and single vision lenses. The SAL lenses do not compromise DCVA.

Previous studies have confirmed the relationship between time spent outdoors and the prevalence of myopia. Outdoor activity is a protective factor against the development of myopia [37]. Of 46 participants, only 7 reported that their hobbies were outdoor activities. Unfortunately, the data on the time and frequency of outdoor activities were not collected in this study. Another risk factor is parental history of myopia. Children with myopic parents had a higher risk of developing myopia [38]. The prevalence of parental myopia was high in this study. 58% of participants had two myopic parents, and 32% of participants had one myopic parent, which were much higher than that in Ip JM et al.’s survey. The possible reasons were the differences in the race, region and inclusion criteria of participates.

Another detail that should be noted was that we did not dispense new equipment, even though the degree of myopia may have progressed at 3 months and 9 months (V3 and V6). One thing that needs to be considered is whether undercorrection will accelerate myopic progression. Previous studies demonstrated that undercorrection produced no significant difference in myopia progression compared with full correction [39, 40]. Chung K et al. reported that undercorrection produced myopia progression [41]. However, the difference between undercorrection and full correction was slight (-0.23 D) over the 24-month research period. We speculated that undercorrection in the short-term would not accelerate the progression of myopia.

One limitation of this study was failure to collect the environmental factors, such as time and frequency of outdoor activities. Environmental factors played an important role in myopia based on several epidemiological studies. But all participants were randomly recruited and assigned to two groups, which could reduce the impact of bias. Another limitation was the lack of information on the prior year’s myopia progression. However, the main purpose of this study was to evaluate the control effect of SAL lenses, rather than to analyze related factors. Further studies should be designed to observe the influence of environmental and prior progression factors on myopia control effect of such spectacle lenses.

## Conclusion

In conclusion, lenses with contiguous aspherical lenslets are an emerging opportunity for myopia control. With the exception of the adaptation problem in the initial stage of using SAL lenses, this type of lens could be well adapted. If SAL lenses are capable of slowing myopia progression, they will provide a safe and effective optical strategy option for children.

## Supplementary Information

**Additional file 1.**

## Data Availability

The data that support the findings of this study are not publicly available because they contain information that could compromise the privacy of research participants, but are available from the corresponding author (Lijie Hou) upon reasonable request.

## Abbreviations

SAL: Slightly aspherical contiguous lenslets technology
Ortho-K: Orthokeratology
PALs: Progressive addition spectacle lenses
AL: Axial length
SER: Spherical equivalent of autorefraction
CT: Corneal thickness
ACD: Anterior chamber depth
LT: Lens thickness
DCVA: Distance-corrected visual acuity
ETDRS: Early treatment diabetic retinopathy study
NPC: Near and far phoria, near point of convergence
SVL: Single vision lenses
