# Supplementary material for: Spectacle lenses with slightly aspherical lenslets for myopia control: clinical trial design and baseline data
Source: BMC Ophthalmol. 2022 Aug 16;22:345. doi: 10.1186/s12886-022-02562-0 (PMC9382742; doi:10.1186/s12886-022-02562-0)
Supplement: Supplementary file 1 — Additional file 1. [file 12886_2022_2562_MOESM1_ESM.docx]

1. What about the vision clarity when you wearing glasses？

1 = blurred, 10 = clear

1. Do you feel ghost images when you wearing glasses？

1 = none, 10 = severe

1. Are you satisfied with the glasses？

1 = not satisfied, 10 = satisfied

1. Do you feel comfortable when you wearing glasses？

1 = uncomfortable, 10 = comfortable

1. How many hours do you wear glasses every day?
2. How many days do you wear glasses every week?
